# *In silico* Analysis of *Pasteurella multocida* PlpE Protein Epitopes As Novel Subunit Vaccine Candidates

**DOI:** 10.29252/ibj.25.1.41

**Published:** 2020-01-04

**Authors:** Saied Mostaan, Abbas Ghasemzadeh, Parastoo Ehsani, Soroush Sardari, Mohammad Ali Shokrgozar, Mohsen Abolhassani, Gholamreza Nikbakht Brujeni

**Affiliations:** 1 Molecular Biology Department, Pasteur Institute of Iran, Tehran, Iran;; 2 Biotechnology Research Center, Pasteur Institute of Iran, Tehran, Iran;; 3 National Cell Bank of Iran, Pasteur Institute of Iran, Tehran, Iran;; 4 Hybridoma Lab, Department of Immunology, Pasteur Institute of Iran, Tehran, Iran;; 5 Department of Microbiology and Immunology, University of Tehran, Iran

**Keywords:** Pasteurella multocida, Polytope, Vaccines

## Abstract

**Background:**

*Pasteurella multocida *is a Gram-negative, non-motile, non-spore forming, and aerobic/anaerobic cocobacillus known as the causative agent of human and animal diseases. Humans can often be affected by cat scratch or bite, which may lead to soft tissue infections and in rare cases to bacteremia and septicemia. Commercial vaccines against this agent include inactivated, live attenuated, and non-pathogenic bacteria. Current vaccines have certain disadvantages such as reactogenicity or reversion to virulence. Therefore, the aim of this study was to reach a multi-epitope vaccine candidate that could be serotype independent and covers most incident serotypes of *P. multocida*.

**Methods:**

In this study, reverse vaccinology strategy was used to identify potentially immunogenic and protective epitopes. First, multiple alignments of different sequences of PlpE from various serotypes of *P. multocida* were analyzed to identify the conserved regions. Bioinformatics tools were then applied to predict and select epitopes for further studies.

**Results:**

Three different conserved immunogenic regions were selected according to the selected criteria, and their various sequential orders were evaluated structurally by *in silico* tools to find the best order.

**Conclusion:**

In searching the epitopes of PlpE to design a new vaccine candidate against pasteurellosis, we found the region 1 + region 2 + region 3 (without any linker between regions) of epitope, including the regions of PlpE protein of *P. multocida*, as the appropriate serotype independent vaccine candidate against pasteurellosis.

## INTRODUCTION

*Pasteurella multocida*, a Gram-negative, non-motile, non-spore forming, aerobic/anaerobic cocobacillus was first observed by Revolee in 1877 and identified in 1880 by Pasteur (reviewed in^[^^1^^]^). This bacterium is the normal flora of the upper respiratory tract and the gastrointestinal tract of domestic and wild animals and birds, which can infect humans and animals^[^^2^^]^.

Existing vaccine candidates against this pathogen may be divided into live attenuated and killed vaccine categories. Live attenuated vaccines have several shortcomings, including the induction of short-term or ineffective immunity^[^^3^^,^^4^^]^, and in particular, the possibility of reversion to virulence in cases where the attenuation mechanism is not defined^[^^5^^]^. However, the effectiveness of the killed vaccines varies from inefficiency to short-term stimulation of immunity, hence providing the protection only against the strain from which the vaccine is prepared^[^^6^^,^^7^^]^.

Considering the limitations of the current vaccines, there is clearly a need for the development of more effective vaccines that are simpler to produce. One such approach is to develop new subunit vaccines, which would have some advantages, such as easier production and prevention of the possible reversion to virulence^[^^8^^]^.

PlpE is an immunogenic Omp protein of *P. multocida*, and mice or chickens immunized with this recombinant protein are protected against challenge with other *P. multocida* serotypes^[^^3^^,^^4^^]^. As a lipid modified, surface-exposed Omp, PlpE is important in complement-mediated killing. We hypothesized that identifying the epitope regions of PlpE protein, which is common to all serotypes, will offer the probability of introducing a vaccine candidate capable of raising protection against all *P. multocida* serotypes.

The aim of the present study was to investigate the potential epitopes of the PlpE protein from *P. multocida* A:1, one of the causative agents of pasteurellosis. The protein was mapped in terms of immunogenicity and also evaluated in view of hydrophilicity, antigenicity, half time, stability and so on. Our data indicated that three different regions, each consisting of one or more epitopes, are responsible for protection. These regions are suitable to express as a possible recombinant subunit vaccine. 

## MATERIALS AND METHODS

To predict the biophysical and immunological characteristics of P1pE, the available amino acid sequences of PlpE proteins were obtained from the National Center for Biotechnology Information (NCBI) database. *P. multocida* was the key term for both nucleotide and protein sequences, and only complete sequences were selected. 

**Multiple alignments**

Alignment procedure was performed using Multiple Alignment tool of Vector NTI Advance (TM) 11.0 software, Invitrogen, USA. Nucleotide and amino acid sequences of 11 different serotypes were entered to identify the conserved regions of PlpE.

**Epitope investigation**

Amino acid sequences of PlpE from different serotypes of *P. multocida* were used as an entry to identify probable epitopes, using Bepipred linear epitope prediction of IEDB. While linear epitopes of PlpE have been reported to be potent enough to raise immune response, B cell epitope prediction tools of IEDB database was used to predict the linear epitopes.

**Antigenicity evaluation**

An antigen is a molecule that binds to Ag-specific receptors or to antibody but cannot necessarily induce an immune response in the body by itself. Therefore, antigenicity and epitope selection are needed to be considered simultaneously. Potentially antigenic regions were identified using the antigenic peptide prediction tool of Immunomedicine Group (http://imed.med.ucm.es/Tools/antigenic.html).

**Hydrophobicity evaluation**

Hydrophilic parts of molecules are exposed to milieu and can be detected by antibodies. Potential hydrophilicity was assessed using the ProtScale tool in ExPASy database (https://web.expasy.org/protscale/).

**The 3D structure design, upgrade, and review**

Linear epitopes of PlpE have been reported to be potent enough to raise the immune response; however, the potential conformational epitopes of PlpE were investigated to determine whether they stimulate more potent immune response. To achieve this goal, the 3D structures were designed. For this purpose, amino acid sequences of different PlpEs were analyzed using the I-TASSER tool and LOMETS method. To upgrade the 3D structures, the energy minimization tool was used, which makes the molecules more similar to the native form. This program enhances the 3D structure of the protein and also promotes the 3D structure of the desired protein. After applying the changes to the secondary structure of the protein, the alpha helix and beta sheets of the proteins were evaluated by WebLab viewer (https://www.scalacs.org/TeacherResources/). Similar changes of amino acids between different PlpEs of the various serotypes of *P. multocida* were considered minor and ignored, whereas dissimilar changes were regarded major, and the region was excluded from our selection.

**Check for repetitive sequences (Tandem repeat)**

Tandem repeats are regions of sequences repeated several times. Each epitope within identical regions were evaluated by LALIGN tool (https://embnet.vital-it.ch/software/LALIGN_form.html). Epitopes with the highest rate of repeatability within PlpE protein sequences are better candidates as they could be detected more frequently by antibodies. 

**Table 1:** Selected *P. multocida* serotype sequences

**Accession number**	**General description**	**Serotype**	**Host**	**Size (bp)**
EF219452	*P. multocida* strain X73 Lipoprotein E (PlpE)	A:1	Chicken	1011
EF219453	*P. multocida* strain p-470 Lipoprotein E (PlpE)	A:1(2009), A:5(2015)	Chicken	1008
EF219454	*P. multocida* strain p-61 Lipoprotein E (PlpE)	A:3	Buffalo	1008
EF219455	*P. multocida* strain p-1059 Lipoprotein E (PlpE)	D:11	Pig	1008
EF219456	*P. multocida* strain p-1662 Lipoprotein E (PlpE)	D:1	Buffalo	1008
EF219457	*P. multocida* strain ATCC 12948 Lipoprotein E (PlpE)	B:2	Cattle	1017
GQ202239	*P. multocida* strain BNM-p52 outer membrane lipoprotein	A:4	Turkey	1008
GQ202240	*P. multocida* strain BNM-194 outer membrane lipoprotein	A:3	Pig	1011
GQ202241	*P. multocida* strain BNM-47 outer membrane lipoprotein	D:3	Pig	1017
GU108958	*P. multocida* strain C48-1 Lipoprotein E (PlpE)	A:3	Turkey	1011

Further information is available based on their accession number in NCBI.

**Solubility evaluation**

Solubility of the potential recombinant protein was predicted by using the Recombinant Protein Solubility Prediction tool (https://biotech.ou.edu). This tool predicts the amount of protein expression in *Escherichia coli*. To calculate the protein half-life, ProtParam tool was used.

**Half-life evaluation**

The half-life of proteins can vary widely and means how long they are accessible to the immune system. This characteristic was predicted using the ProtParam tool (https://web.expasy.org/protparam/) of the server Expasy.

**Antibody evaluation**

For the evaluation of the antibody, the 3D structure of PlpE protein and selected regions was docked by Hex software (8.0.0 for windows) against 100 antibodies, which their 3D structure was available online (https://www.rcsb.org/). 

## RESULTS

**Conserved regions**

**Fig. 1:**
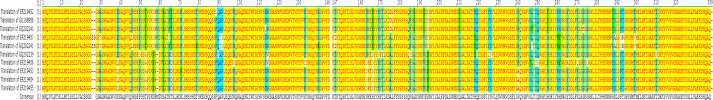
Alignment of 10 different PlpE proteins of *P. multocida*. Yellow regions show the conserved regions, and other colors indicate the regions of differences

Conserved and divergent regions of 10 different PlpEs (Table 1) were evaluated, and the conserved or similar regions were selected for further evaluations (Fig. 1). Most of the changes were point mutation, but insertions were observed in positions 26 and 27. In the 59^th^ position, there was a deletion, and the biggest change was found to be at positions 89-92.

**Epitope mapping and antigenicity evaluation**

**Fig. 2:**
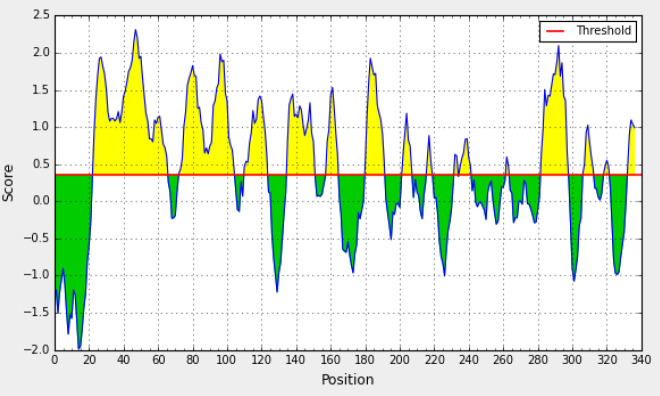
Linear epitope prediction of *P. multocida* PlpE. Possible epitopes indicated in yellow and green zones are not potent enough to raise the immune response of the host

**Fig. 3:**
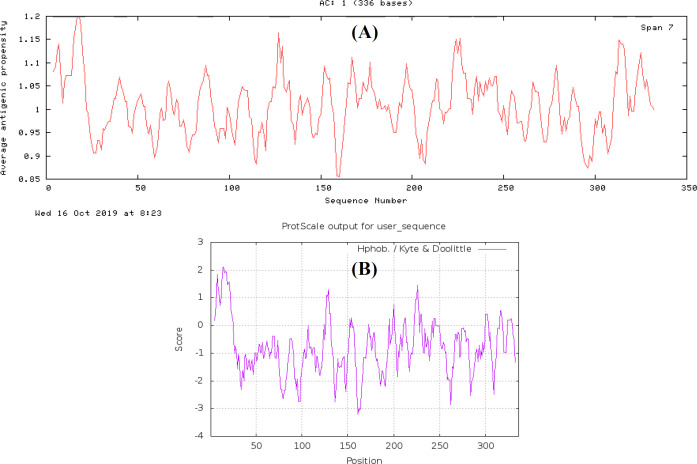
Antigenicity (A) and hydophobicity (B) evaluation of *P. multocida* PlpE

All the possible epitopes of the 11 selected PlpEs of *P. multocida* were investigated, and linear epitopes were predicted and selected (Fig. 2). Many small potential epitopes were achieved, but none of them was suitable to clone and express. Therefore, some more conserved regions consisting of multiple epitopes were selected as candidate regions, and then further evaluated to check their compliance. The possible antigenicity of the 10 selected PlpEs of *P. multocida* was evaluated, and all antigenic regions were identified (Fig. 3A). The same as the epitopes, various antigenic regions were obtained. Candidate conserved regions were comprised of multiple epitopes overlayed on potential antigenic regions. 

**Hydrophobicity evaluation**

Hydrophilic and hydrophobic regions of the 11 selected PlpEs of *P. multocida* were identified and presented in Figure 3B. Based on different evaluations mentioned above on 10 different PlpEs, three regions were of our interest as immuno-dominant regions (Table 2).

**Construction of polytope order**

Three selected regions came together in different sequential orders to make our polytopes. Six different polytopes were established based on their orders in a linear fashion. After the virtual construction of polytopes, they were mapped again for epitopes, and due to the accessibility of regions separately by antibodies, it was decided not to put a linker between the regions.

**Solubility and half-life evaluation**

The predicted solubility of different *P. multocida* PlpEs and various polytopes were evaluated. All PlpEs and polytopes were insoluble except for region 3, which was predicted to be 100% soluble. The predicted half-life of different *P. multocida* PlpEs and fusion proteins was also assessed. All the PlpEs were stable, while the most stable fusion proteins were found to be the polytope containing region 1 plus regions 2 and 3, in a sequential order. The main epitope within region 3 was very unstable in terms of half-life, but still 100% soluble. 

**Potential detection of PlpE by immune system**

The Selected regions of PlpE could be detected by antibodies similar to the intact protein, which indicates the appropriate accessibility of selected regions to examined antibodies.

## DISCUSSION

Immunization is one of the most effective tools for the prevention of pasteurellosis. The use of recombinant vaccines encoding individual epitopes linked together in a linear ‘string-of-beads’ fashion has been a successful strategy for the simultaneous delivery of multiple epitopes. The polytope approach has the added advantage of avoiding potential hazards associated with immunizing with full-length antigens. Since it has been reported that each epitope within the construct may be individually immunogenic, a polytope approach is attractive since it directs a broad response simultaneously against multiple epitopes which appears to be crucial for the development of effective vaccines against several diseases. The first generation of vaccines against this disease was consisted of formalin-killed bacteria that are still available and can elicit protection against homologous infection in different animal species^[^^9^^]^. Heat-killed vaccines have been indicated to stimulate the partial protection in comparison to the formalin killed ones^[^^10^^]^, suggesting that the main protective epitopes of *P. multocida *are heat-labile. Development of bacterial vaccines from different strains of *P. multocida* have some disadvantages, including the lack of full cross-protection, short-term immunity, and induction of local inflammation at the site of injection^[^^11^^]^.

**Table 2:** Specifications of selected regions

**Region number**	**Amino acid location**	**Sequence**
1	23-104	GGGGSAGNRADRVEEKAQPVQSNSEPSSTPIKHPMTNSATNTSLHDKLSMSSHDTSKENSQQSSL QAPLEQEKNQPAQENLT
2	135-202	YNSQYNDDPVFDKTKTQSKTISLVDGKNENKEHYYHFTLKDDLFYYGSYGQPSSDYKKIEENYIYAIK
3	262-321	HNRGYDDLFKLSGEGRNLILTPHKNNPYDLSPTGPDNMTMELNFINAEKTDKKYVVGVGK

*P. multocida* is attenuated by incubation with mutagens and streptomycin or subjected to gene deletion to produce avirulent live strains. Unlike pepsin-treated vaccine, both the heat-inactivated bacteria and the bacterial ghosts are able to develop protection. This fact confirms that the main protective antigens of the *P. multocida* are proteins located in the cells wall^[^^12^^]^. To produce cheaper large-scale vaccines, DNA vaccines were evaluated, and DNA-encoding *P. multocida* toxin, OmpA, OmpH, and PlpE were tested. Although these vaccine candidates elicited both humoral and cellular immunity, they were not protective in monovalent form unless introduced as combined forms^[^^13^^]^. DNA vaccines have shown some potential prospects, but their use is limited. Some shortcomings like the risk of affecting genes involved in the cell growth, possibility of inducing antibody production against DNA, possibility of tolerance to the antigen (protein) produced, and potential for atypical processing of bacterial and parasite proteins have been speculated, which restrict the development of such vaccines^[^^14^^]^. Subunit vaccines offer a safer alternative and benefit from defined components, simple large-scale production, and the ability to modify and improve proteins^[^^15^^]^. 

Recently, capsular polysaccharide, lipopoly-saccharide, siderophores, *P. multocida* toxin, dermonecrotic toxin, and PlpE of *P. multocida* have been indicated to elicit high antibody titers^[^^16^^]^. Hatfaludi *et al.*^[^^17^^]^ have reported an extensive set of Omp and outer membrane-associated proteins through the bioinformatics analysis of the *P. multocida* genome. Due to their predicted localization as secreted- or surface-exposed proteins, they were proposed to have the potential to elicit a protective immune response. Until now, 71 of these proteins were tested for immunogenicity and for their ability to protect against lethal *P. multocida* infection. Only a single recombinant protein, PlpE, was capable of eliciting the protective immune response^[^^17^^]^. Wu *et al.*^[^^3^^]^ have already described PlpE as a potential candidate vaccine. Okay *et al.*^[^^18^^]^ have tested recombinant OmpH, PlpE, and PlpEC-OmpH fusion proteins formulated with oil-based CpG Oligodeoxynucleotide adjuvants. The recombinant proteins PlpE and PlpEC-OmpH fusion stimulated 80% and 60% protection, respectively. Their findings indicated that the recombinant PlpE was a possible acellular vaccine candidate^[^^18^^]^. Based on these facts, PlpE was selected for evaluation in our study. 

According to the findings of our bioinformatics studies, three different conserved regions of PlpE fulfilled the selected criteria, i.e. immunogenicity, antigenicity, different serotypes coverage, half-life, and antibody accessibility. Conserved region 1 comprising of amino acids 23-104 of PlpE, conserved region 2 with amino acids 135-202, and conserved region 3 with amino acids 262-321 are hypothesized as the most promising regions of PlpE to confer protection against *P. multocida*. The best polytope order was found as region 1, region 2, and region 3. Further studies are underway to clone/express these conserved regions and evaluate their efficacy as vaccines against infection by *P. multocida*.
